# Supplementary material for: Effect of a New Tele-Rehabilitation Program versus Standard Rehabilitation in Patients with Chronic Obstructive Pulmonary Disease
Source: J Clin Med. 2021 Dec 21;11(1):11. doi: 10.3390/jcm11010011 (PMC8745243; doi:10.3390/jcm11010011)
Supplement: Supplementary file 1 [file jcm-11-00011-s001.zip › jcm-1430410-supplementary.pdf]

Supplemental Digital Content to the Article:

Report 1 (Exclusion criteria) 1

Report 2 (Reasons for dropout) 2

Report 3 (Participants non participants) 3

Report 4 (Baseline data) 4

Report 5 (Follow-up data) 6

Report 6 (Confidence interval of other variables) 18

Report 7 (Non inferiority 8 weeks vs 3- and 6 months follow-up) 20

Report 8 (Exercise Time, Adherence and Patient Satisfaction in the Telerehabilitation with VAPA group) 22

Report 1 (Exclusion criteria)

1. Severe comorbidities precluding training such as i.e.
    a. Unstable heart disease
    b. Dysregulated diabetes
    c. Known malignant disease
2. Any other illness making the patient inappropriate for participating in the study
3. Non-compliant patient
4. Severe vision or hearing impairment
5. Non-Danish speaking
6. Unwillingness or inability to follow the protocol
7. COPD exacerbation in the preceding 6 weeks.

Report 2 (Reasons for dropout)

- Personal causes 7
- Worsening condition 5
- Not following training protocol 4
- Withdraw consent 10
- Transportation problems 2
- Had an accident 1
- Sensor problems 1
- Dead 1

Report 3 (Participants non participants)

Differences between participants and non participants

Table S1. Baseline parameters between participants and non-participants.

There was only a significant difference in FEV1/FVC ratio% between participants and non-participants.

	NON-PARTICIPANTS				PARTICIPANTS				P-value	Mean Difference
	**Mean**		**SD**	**n**	**Mean**		**SD**	**n**		
Age	69.57	±	7.73	21	69.97	±	9.18	54	0.86	-0.40
6MWT*	395.45	±	90.85	11	376.23	±	92.02	53	0.53	19.23
FVC**	2.30	±	0.87	20	2.24	±	0.72	53	0.76	0.06
FVC Predicted%	66.75	±	16.40	20	68.79	±	18.81	53	0.67	-2.04
FEV1***	1.02	±	0.62	21	0.88	±	0.33	53	0.22	0.14
FEV1 Predicted%	0.43	±	0.12	17	1.03	±	4.48	53	0.99	0.05
FEV1_Ratio	0.43	±	0.12	17	4.48	±	0.62	53	0.59	-0.60
FEV1_Ratio%	53.00	±	14.23	20	43.853	±	17.02	54	**0.04**	9.15
Male/female	11 male / 10 female				31male / 23 female				0.69	#

* 6 Minute Walk Test. ** Forced Vital Capacity *** Forced expiratory volume in one second
# According to the Pearson chi-square test the gender doesn't differ between groups.

Report 4 (Baseline data)

Baseline data

Table S2 Baseline data of all randomized patients

		All patient (*n*=54)
**Male, n (%)**		31 (57.40%)
**Age (years), mean (SD)**		69.96 (9.18)
**Smoking status**	*Current, n (%)*	7 (15.2%)
	Former, n (%)	39 (84.8%)
	Never, n (%)	0 (0%)
**Long-term oxygen therapy, n (%)**		4 (7.4%)
**FVC (% predicted), mean (SD)**		68.79 (18.81)
**FEV1 (% predicted), mean (SD)**		34.53(11.67)
**FEV1_Ratio%**		43.85 (17.02)
**6MWD (m), mean (SD)**		376.23 (92.02)

SD: Standard deviation; FVC: Forced vital capacity; FEV1: Forced Expiratory Volume in the first second; 6MWD: distance walked during the 6-minute walk test

Table S3 Baseline demographics of patients in the control and intervention group

Baseline data shows that participants in the intervention group was younger (5 years, p=0.04), had higher FEV1/FVC Ratio (9.41%, p=0.04), had higher Borg dyspnea CR10 score at 6 minute walk tests (1.03, p=0.05), slightly worst quality of life with respect to SGRQ Activity (0.75%, p=0.05) but better total SGRQ (5,07%, p=0.03)).

Table S3: Baseline demographics in the control and intervention group

BASELINE	CONTROL BL			INTERVENTION BL			Mean Difference	p-value
	N	Mean	Std. Deviation	N	Mean	Std. Deviation		
Age	27	72.49	7.45	27	67.45	10.17	5.04	**0.04**
6MWD	26	366.62	97.77	27	385.48	86.95	-18.87	0.46
Borg CR10 Scale before test	25	1.62	1.17	27	1.79	1.75	-0.17	0.69
Oxym before test	26	94.96	1.99	27	94.30	2.54	0.67	0.29
HR before test	26	89.65	12.68	27	83.30	16.13	6.36	0.12
4 min gait test	20	3.89	1.28	27	4.08	1.12	-0.18	0.61
Oxym after test	26	88.85	4.93	27	89.74	5.11	-0.89	0.52
HR after test	26	110.92	19.15	27	111.41	22.32	-0.48	0.93
Borg CR10 Scale after test	24	6.58	1.44	27	7.61	2.10	-1.03	**0.05**
Oxym at 7 minutes	26	92.23	4.58	26	92.69	3.89	-0.46	0.70
HR at 7 minutes	26	106.73	16.98	26	103.62	17.07	3.12	0.51
Oxym at 8 minutes	16	94.19	3.67	26	94.50	2.82	-0.31	0.76
HR at 8 minutes	16	101.75	16.74	26	98.69	15.91	3.06	0.56
FVC	26	2.28	0.77	27	2.2104	0.68	0.06	0.75
FVC%_Predicted	26	70.19	17.90	27	67.44	19.89	2.75	0.60
FEV1	26	0.81	0.31	27	.943	0.35	-0.13	0.15
FEV1%_Predicted	26	32.84	8.48	27	36.15	14.06	-3.31	0.31
FEV1_Ratio	26	1.64	6.40	27	.4363	0.13	1.20	0.33
FEV1_Ratio%_Predicted	27	39.15	17.50	27	48.56	15.43	-9.41	**0.04**
Total_weekly_steps	27	9234.37	7126.44	26	8600.77	4831.39	633.60	0.71
Vector_Magnitude (minutes)	27	358.29	261.96	26	282.08	132.88	76.20	0.19
GAD7	27	5.96	6.601	27	3.26	3.859	2.70	0.41
SGRQ_Symptoms	27	61.2064	23.45995	27	56.1459	21.10210	5.06	0.85
SGRQ_Activity	27	76.7948	15.36086	27	77.5419	14.35142	-0.75	**0.05**
SGRQ_Impacts	27	51.2459	15.95986	27	42.8443	15.47139	8.40	0.18
SGRQ_Total	27	60.6433	14.07582	27	55.5693	13.53181	5.07	**0.03**
IADL SCORE	27	2.19	2.288	27	1.11	1.086	1.07	0.46

FVC: Forced vital capacity; FEV1: Forced Expiratory Volume in the first second; 6MWD: Distance walked during the 6-minute walk test; HR: Heart rate; Oxym: Oximetria; 7dVMCPM: 7 days vector magnitud counts per minute; SGRQ: Saint George Respiratory Questionnaire; IADL SCORE: Instrumental Activities Of Daily Living Scale; GAD7: General Anxiety Disorder-7 Questionnaire.

Report 5 (Follow-up data)

Extra analysis for the 8 weeks follow-up.

All patients who completed pulmonary rehabilitation online or in presence were considered for the 8 weeks test analysis. Non-inferiority test was made to check if the TR with VAPA was non-inferior to the standard treatment.

Table S4. Data at follow-up after 8 weeks of training. Analyses are shown with an independent t-test within groups.

**8 WEEKS**	**CONTROL**			**TR WITH VAPA**			Mean Difference	Std. Error Difference	95% Confidence Interval of the Difference		p-value
	N	Mean	Std. Deviation	n	Mean	Std. Deviation			Lower	Upper	
6MWT (m)	17	387.15	100.91	18	434.56	84.49	47.41	31.39	-16.46	111.27	0.14
GAD7	14	4.86	6.75	18	1.83	2.38	-3.02	1.71	-6.51	0.46	0.09
SGRQ_Symptoms	14	48.75	14.83	18	52.67	17.35	3.92	5.81	-7.95	15.79	0.51
SGRQ_Activity	14	80.38	17.57	18	77.57	13.71	-2.81	5.52	-14.09	8.47	0.61
SGRQ_Impacts	14	40.65	20.34	18	37.02	13.50	-3.63	5.99	-15.86	8.60	0.55
SGRQ_Total	14	54.04	16.27	18	51.91	10.59	-2.13	4.76	-11.85	7.59	0.66
IADL SCORE	14	1.14	1.03	18	0.78	0.94	-0.37	0.35	-1.08	0.35	0.30

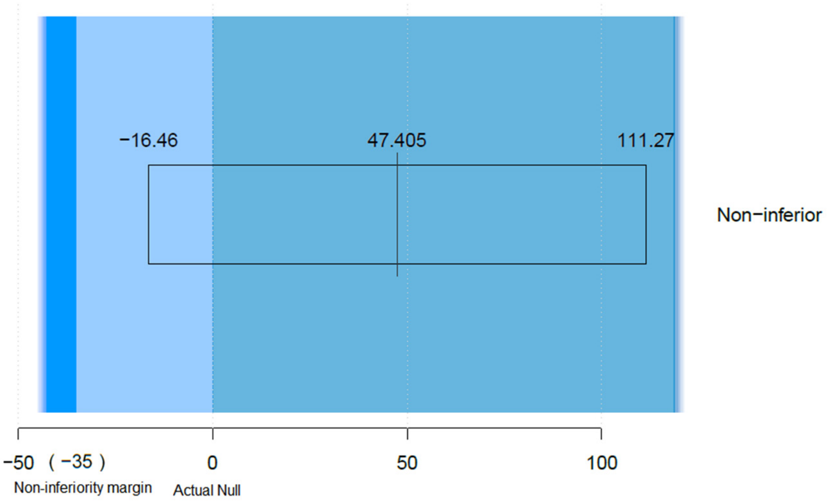

Figure S1. Mean difference and 95% CI for 6MWT between patients in the control and TR with VAPA groups, after 8 weeks of training.

Extra analysis for the 3 months follow-up

Table S5. Data at follow-up three months after completion of training. Analyses are shown with an independent t-test within groups.

**3 M f.u**	**CONTROL**			**TR WITH VAPA**			Mean Differen ce	Std. Error Differen ce	95% Confidence Interval of the Difference		p-value
	n	Mean	Std. Deviation	n	Mean	Std. Deviation			Lower	Upper	
6MWT	13	373.72	125.074	16	429.69	78.215	55.96	37.99	-21.98	133.91	0.15
GAD7	13	3.77	4.76	16	3.00	5.59	-0.77	1.96	-4.78	3.24	0.70
SGRQ_Symptoms	13	47.90	20.33	16	47.59	16.36	-0.31	6.81	-14.28	13.65	0.96
SGRQ_Activity	13	74.83	18.02	16	72.04	17.63	-2.79	6.65	-16.44	10.85	0.68
SGRQ_Impacts	13	36.41	16.50	16	37.33	15.61	0.93	5.98	-11.34	13.19	0.88
SGRQ_Total	13	49.96	13.65	16	49.56	11.85	-0.41	4.74	-10.12	9.31	0.93
IADL SCORE	13	0.85	0.90	16	0.81	0.98	-0.03	0.35	-0.76	0.69	0.92

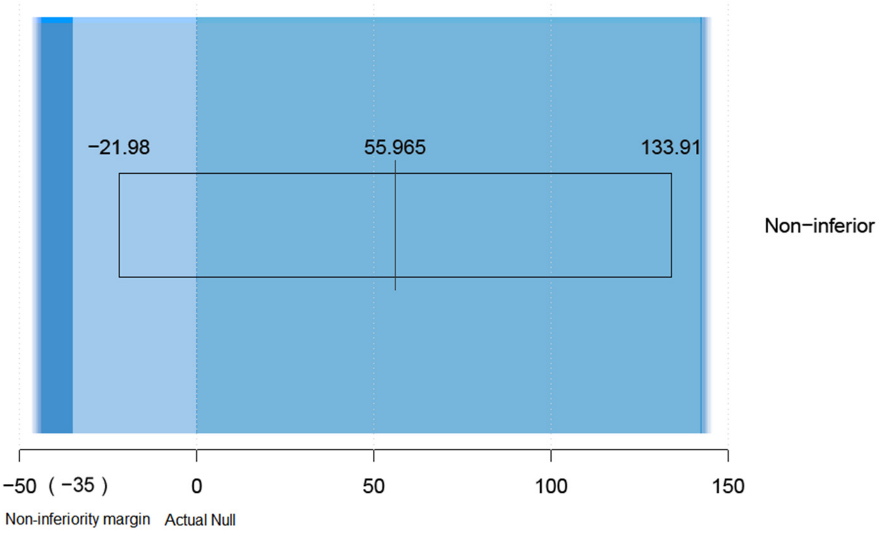

Figure S2. Mean difference and 95% CI for 6MWT between patients in the control and TR with VAPA groups, 3 months after completion of training.

Extra analysis for the 6 months follow-up

Table S6. Data at follow-up six months after completion of training. Analyses are shown with an independent t-test within groups.

6M f.u	CONTROL			TR WITH VAPA			Mean Difference	Std. Error Difference	95% Confidence Interval of the Difference		p-value
	n	Mean	Std. Deviation	n	Mean	Std. Deviation			Lower	Upper	
6MWT	8	320.38	106.84	15	415.60	107.89	95.23	47.08	-2.68	193.13	0.06
GAD7	8	4.50	7.80	15	2.87	5.72	-1.63	2.84	-7.54	4.27	0.57
SGRQ_Symptoms	8	49.53	15.37	15	43.54	19.08	-5.99	7.85	-22.31	10.33	0.45
SGRQ_Activity	8	78.19	12.14	15	75.73	17.63	-2.46	7.01	-17.04	12.11	0.73
SGRQ_Impacts	8	35.20	14.59	15	35.55	12.38	0.35	5.76	-11.63	12.33	0.95
SGRQ_Total	8	50.61	11.51	15	49.06	11.73	-1.56	5.10	-12.17	9.06	0.76
IADL SCORE	8	1.00	0.93	15	0.87	1.25	-0.13	0.50	-1.18	0.91	0.79

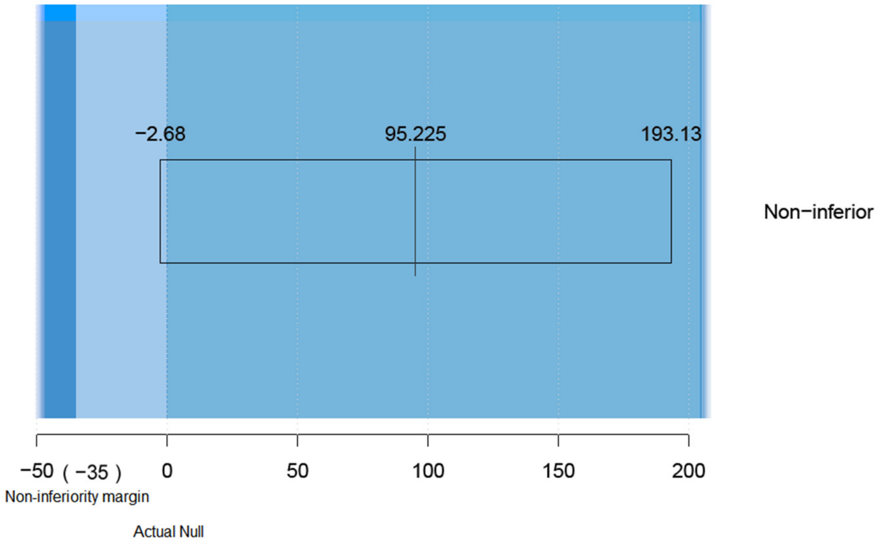

Figure S3. Mean difference and 95% CI for 6MWT between patients in the control and TR with VAPA groups, 6 months after completion of training.

Figures S4 to 11 show the mean over time for each main variable in the control and intervention group making it possible to get an idea of the behavior of the groups over time.

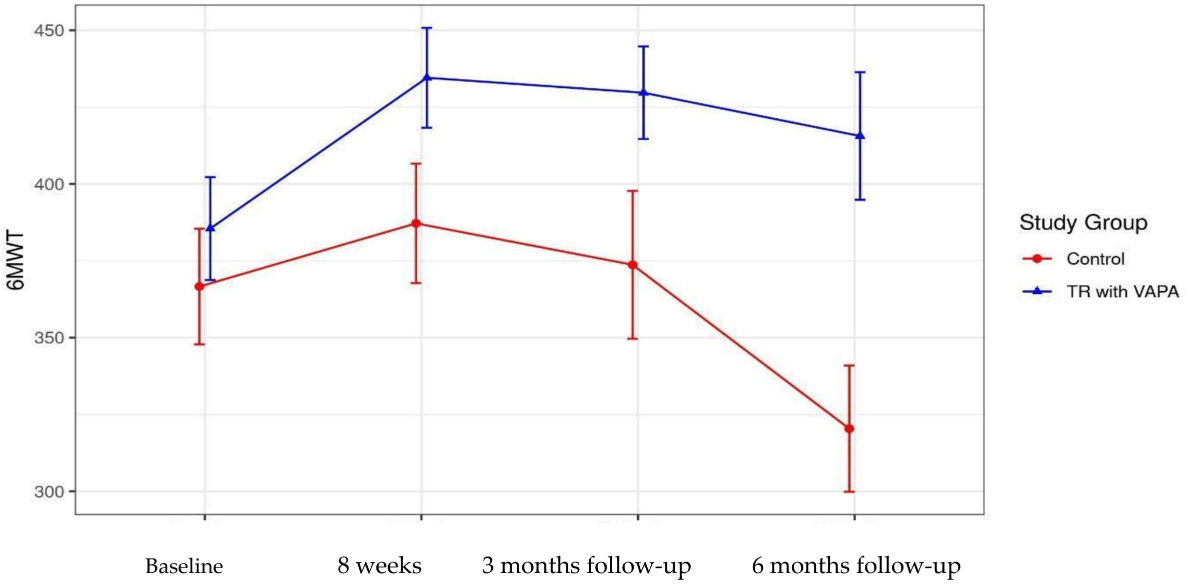

Figure S4 – Mean and Standard Deviation for the 6 minutes walking test over time for patients in the control and intervention group (meters).

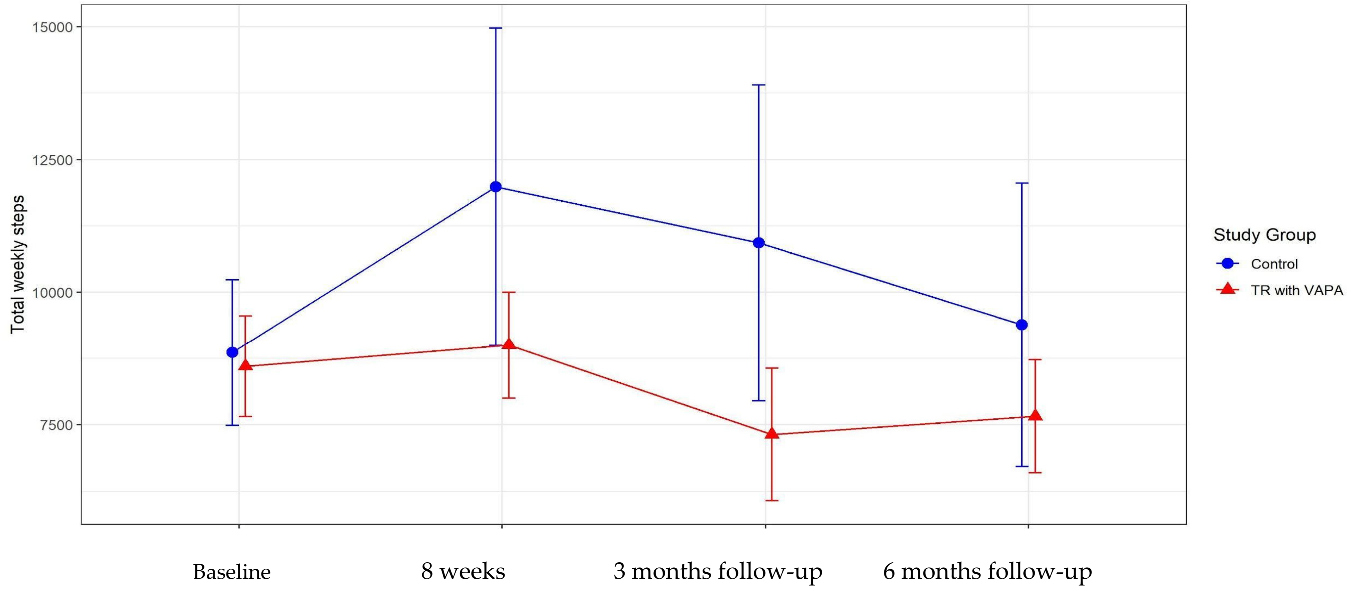

Figure S5. Mean and Standard Deviation for the 7 days pedometer over time for patients in the control and intervention group (meters).

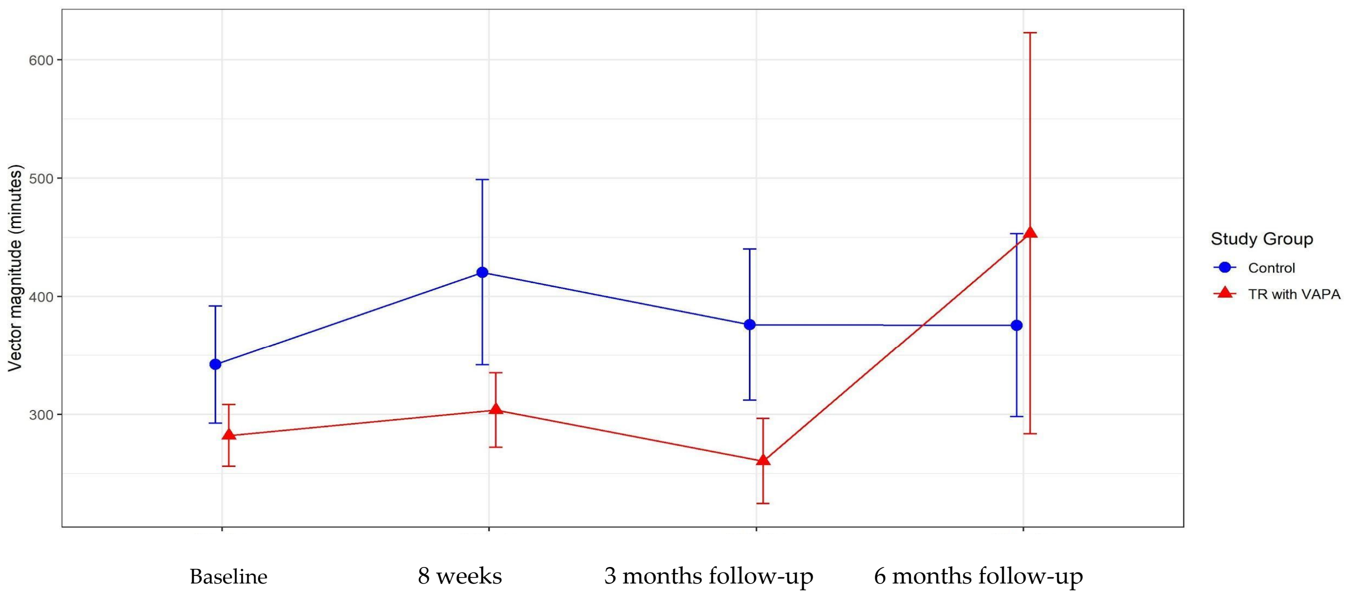

Figure S6. Mean and Standard Deviation for the 7 dVMCPM over time for patients in the control and intervention group (meters).

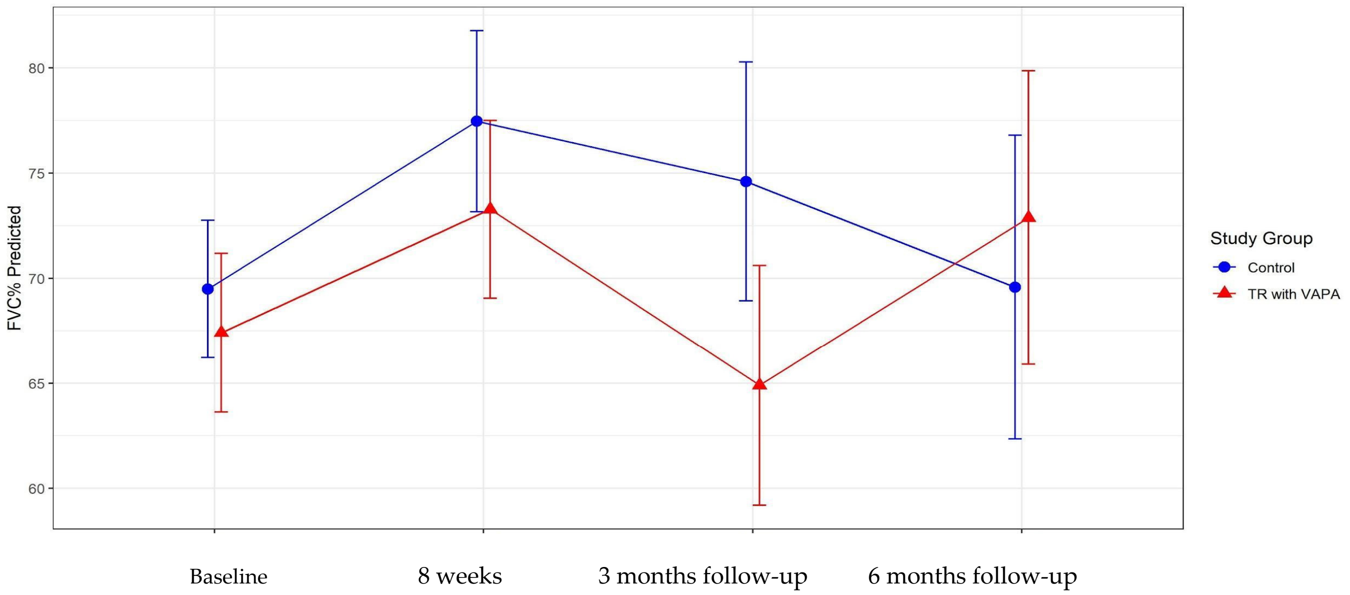

Figure S7. Mean and Standard Deviation for the pulmonary function FVC% over time for patients in the control and intervention group (percentage).

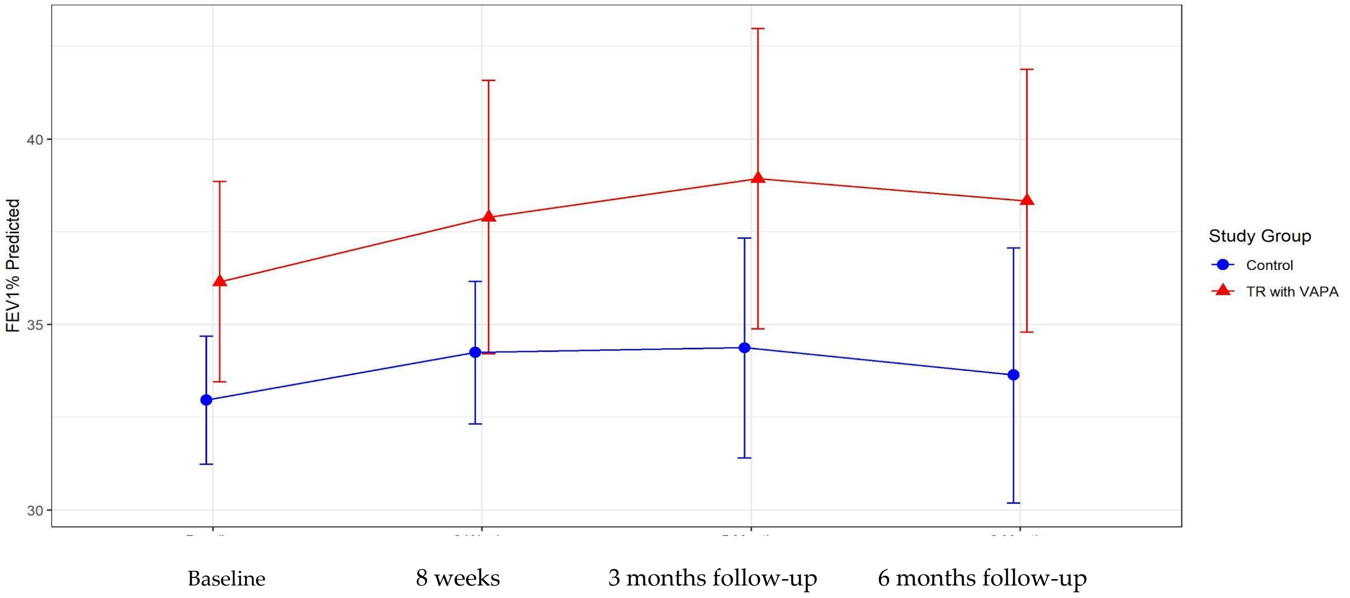

Figure S8. Mean and Standard Deviation for the pulmonary function FVE1% over time for patients in the control and intervention group (percentage).

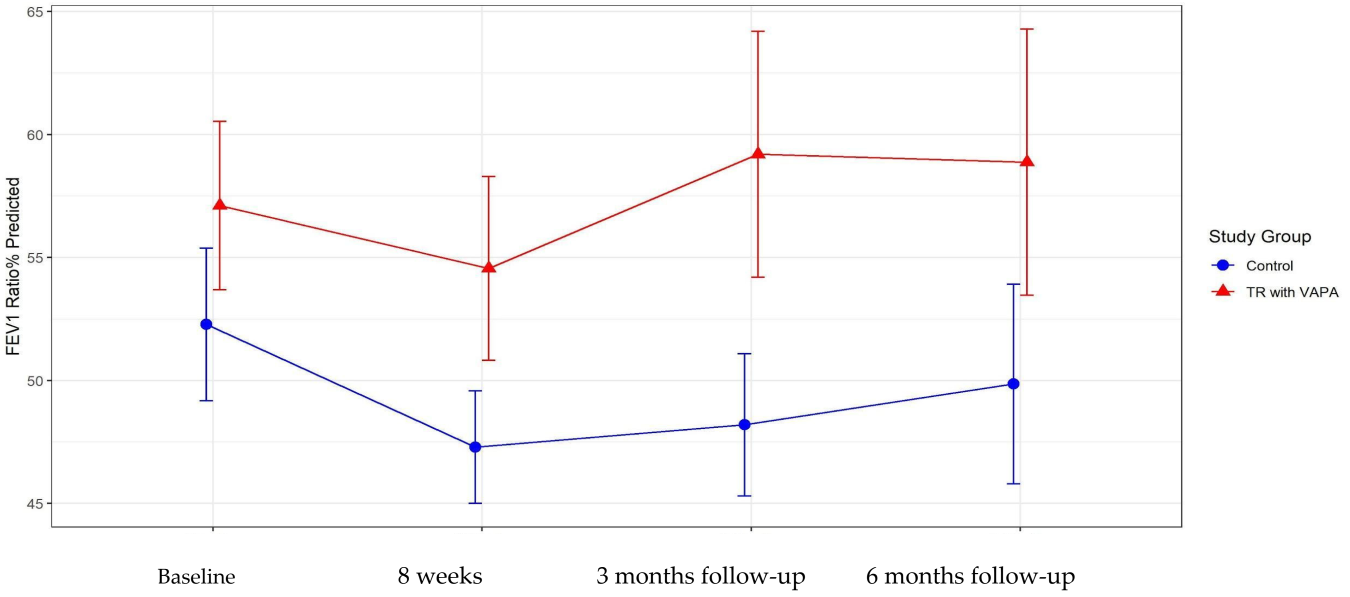

Figure S9. Mean and Standard Deviation for the pulmonary function FVE1/FVC ratio over time for patients in the control and intervention group (percentage).

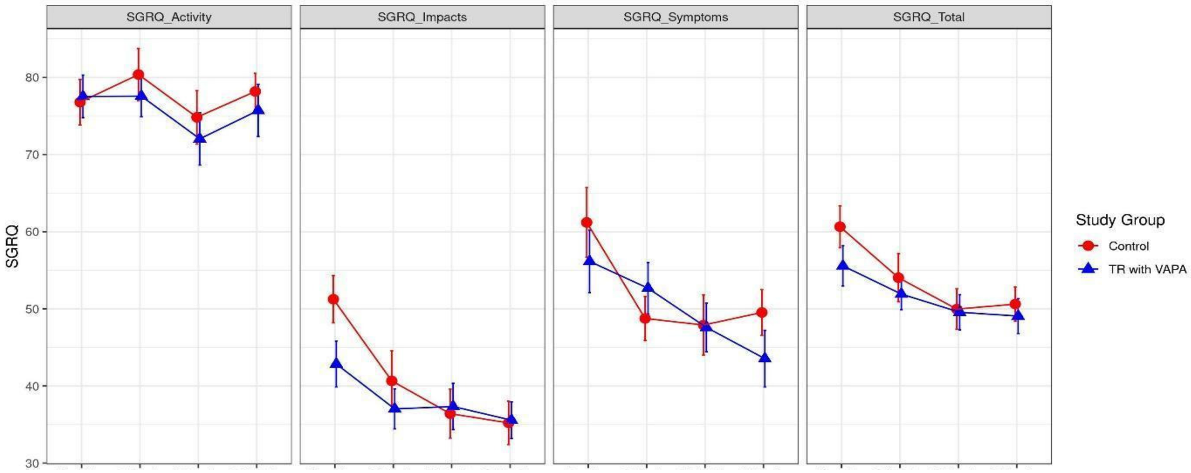

Baseline 8 weeks 3M f.u 6M f.u Baseline 8 weeks 3M f.u 6M f.u Baseline 8 weeks 3M f.u 6M f.u Baseline 8 weeks 3M f.u 6M f.u

Figure S10. Mean and Standard Deviation for the SGQR over time for patients in the control and intervention group.

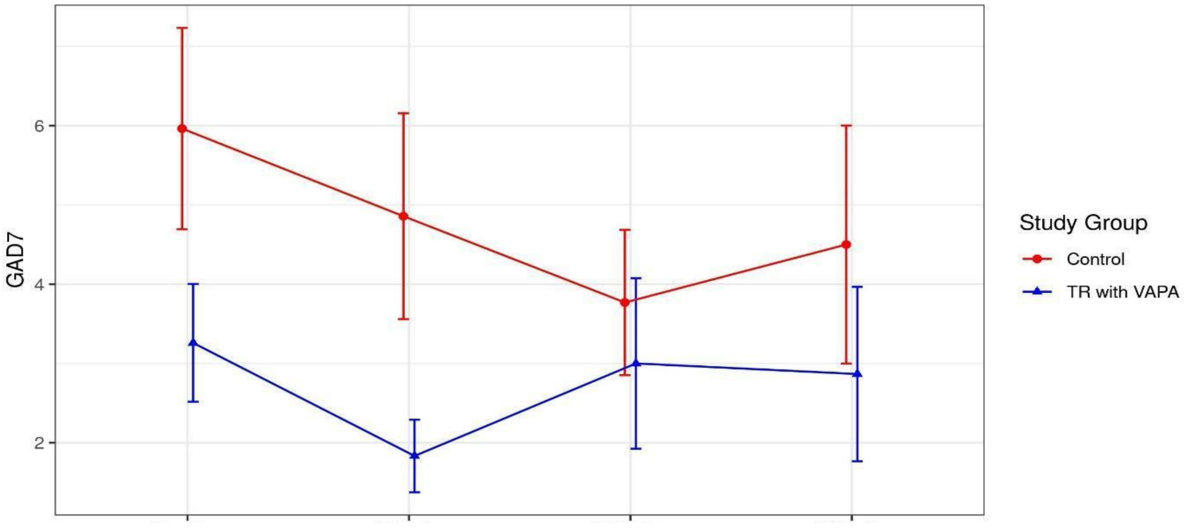

Baseline 8 weeks 3 months follow-up 6 months follow-up

Figure S11. Mean and Standard Deviation for GAD-7 over time for patients in the control and intervention group.

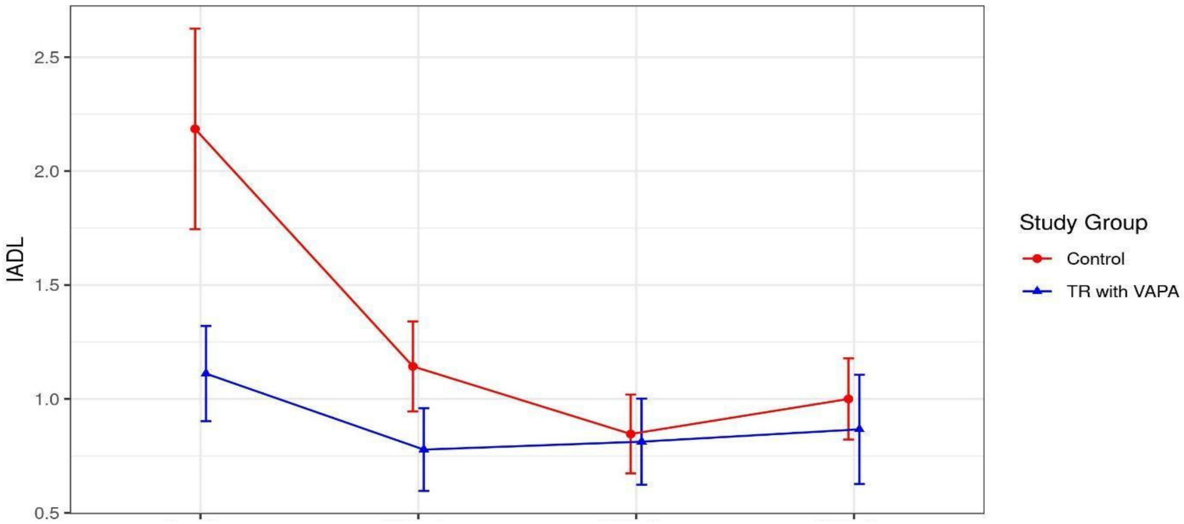

Baseline 8 weeks 3 months follow-up 6 months follow-up

Figure S12. Mean and Standard Deviation for the Instrumental Activities Of Daily Living Scale over time for patients in the control and intervention group.

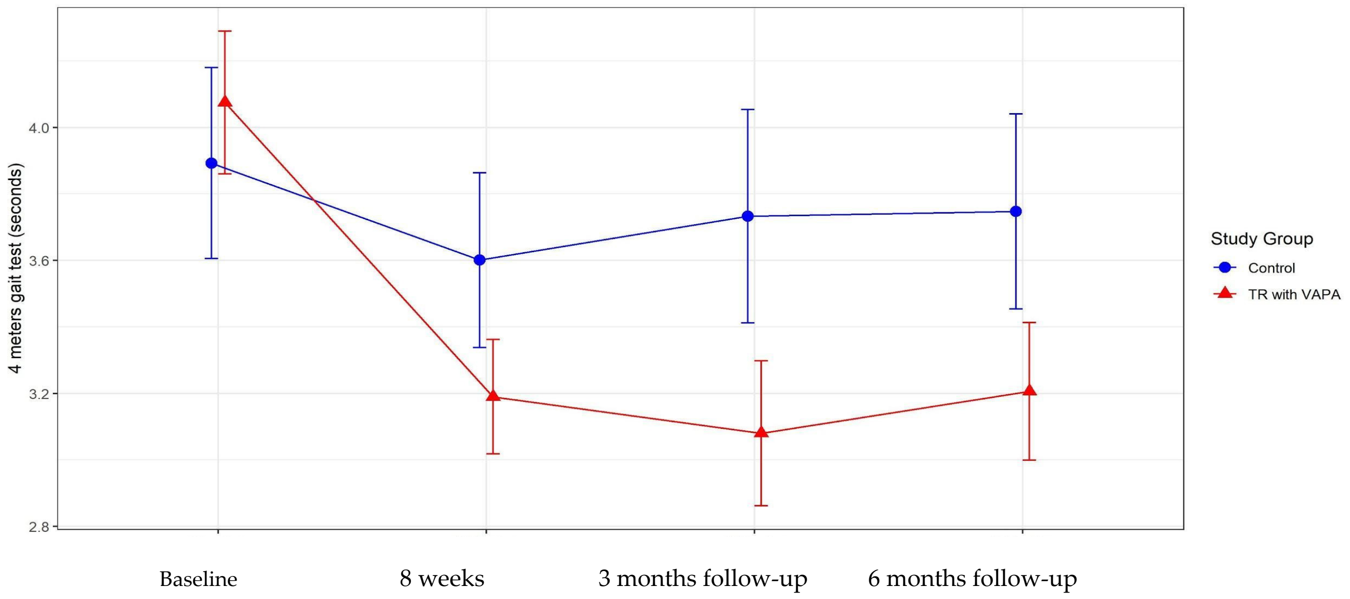

Figure S13. Mean and Standard Deviation for the 4 meters gait test over time for patients in the control and intervention group.

Follow-up data regarding superiority

The control and intervention group were analyzed comparing the baseline with 8 weeks of rehabilitation, 3- and 6 month follow-up in order to verify the behavior of the patients that initiated the training until the end of the trial.

Control group

Table S7 shows the results for the main variables in pulmonary function, physical performance, physical activity, exercise recovery and quality of life in the control group.

Table S7: Analysis of changes over time in pulmonary function, physical performance, physical activity, exercise recovery and quality of life in the control group. (P-value for baseline compared with 8 weeks, baseline compared with 3 months and baseline compared with 6 months)

	**Baseline**			**8 weeks**			**3 M f.u**			**6M f.u**			**p-value**<br>BL-8W<br>BL-3M<br>BL-6M
	**Mean**		**SD**	**Mean**		**SD**	**Mean**		**SD**	**Mean**		**SD**	
FVC, percent predicted	71.76	±	18.28	77.47	±	17.81	76.09	±	17.62	72.25	±	19.24	**0.03**<br>0.28<br>0.25
FVE1%	34.24	±	7.93	32.24	±	8.33	34.36	±	9.82	33.63	±	9.72	0.09<br>0.69<br>0.52
FVE1 Ratio %	40.29	±	9.32	36.71	±	6.86	33.00	±	15.77	43.50	±	9.13	0.11<br>0.30<br>**0.03**
6MWTD	362.76	±	103.13	387.15	±	100.91	373.72	±	125.07	320.38	±	106.84	**0.02**<br>0.36<br>0.93
7 days pedometer	11322.38	±	8465.89	11984.62	±	10782.97	10928.33	±	10304.09	9384.00	±	7068.35	0.58<br>0.70<br>0.27
7 d VMCPM	420.15	±	213.24	420.44	±	282.68	431.05	±	336.95	375.64	±	205.20	0.99<br>0.96<br>0.47

Desaturation	89.35	±	4.50	88.76	±	6.34	90.46	±	4.10	93.88	±	2.10	0.60<br>0.34<br>0.35
Difference between restitution 2min HR	7.42	±	0.89	3.13	±	15.80	13.47	±	0.97	13.03	±	0.32	0.10<br>0.20<br>0.26
SGRQ total	60.37	±	16.28	54.04	±	16.27	49.96	±	13.65	50.61	±	11.51	**0.04**<br>**0.01**<br>**0.04**
IADL SCORE	1.86	±	1.96	1.14	±	1.03	1.85	±	2.03	1.00	±	0.93	**0.04**<br>0.07<br>0.09
GAD-7	5.79	±	7.36	4.86	±	6.75	6.15	±	7.53	4.50	±	7.80	0.21<br>0.24<br>0.40

SD: Standard deviation; FVC: Forced vital capacity; FEV1: Forced Expiratory Volume in the first second; 6MWD: Distance walked during the 6-minute walk test; HR: Heart rate; Oxym: Oximetria; 7dVMCPM: 7 days vector magnitud counts per minute; SGRQ: Saint George Respiratory Questionnaire; IADL SCORE: Instrumental Activities Of Daily Living Scale; GAD7: General Anxiety Disorder-7 Questionnaire.

Intervention group

Table S8 shows the results for the main variables in pulmonary function, physical performance, physical activity, exercise recovery and quality of life in the control group.

Table S8: Analysis of changes over time in pulmonary function, physical performance, physical activity, exercise recovery and quality of life in the intervention group. (P-value for baseline compared with 3 months, baseline compared with 6 months and baseline compared with 9 months)

	**Baseline**			**8 weeks**			**3 M f.u**			**6M f.u**			**p-value**<br>BL-8W<br>BL-3M
	**Mean**		**SD**	**Mean**		**SD**	**Mean**		**SD**	**Mean**		**SD**	BL-6M
FVC, percent predicted	71.56	±	18.93	73.28	±	17.84	65.40	±	24.68	73.07	±	16.85	0.39<br>0.30

													0.43
FVE1%	35.67	±	13.75	37.89	±	15.67	38.93	±	15.69	38.33	±	13.73	0.39<br>0.10<br>0.26
FVE1 Ratio %	45.39	±	14.45	48.00	±	14.84	51.13	±	16.30	50.47	±	14.21	0.07<br>0.05<br>0.16
6MWTD	390.61	±	88.84	434.56	±	84.49	429.69	±	78.21	415.60	±	107.89	**0.03**<br>0.07<br>0.30
7 days pedometer	8834.50	±	4387.07	8999.78	±	4241.69	7318.94	±	4983.98	7660.82	±	3986.32	0.87<br>0.27<br>0.19
7 d VMCPM	294.38	±	131.22	303.72	±	133.46	260.42	±	143.87	453.39	±	634.93	0.77<br>0.40<br>0.41
Desaturation	90.50	±	4.49	91.78	±	4.76	91.13	±	4.66	91.33	±	5.46	0.14<br>0.66<br>0.46
Difference between restitution 2min HR	8.70	±	3.83	19.34	±	4.14	15.56	±	0.57	12.00	±	0.60	0.75<br>0.74<br>0.16
SGRQ total	55.18	±	13.60	51.91	±	10.59	49.56	±	11.85	49.06	±	11.73	0.12<br>0.11<br>0.25
IADL SCORE	1.17	±	1.10	0.78	±	0.94	0.81	±	0.98	0.87	±	1.25	**0.04**<br>0.22<br>0.67
GAD-7	2.78	±	2.94	1.83	±	2.38	3.00	±	5.59	2.87	±	5.72	**0.02**<br>0.75<br>0.73

SD: Standard deviation; FVC: Forced vital capacity; FEV1: Forced Expiratory Volume in the first second; 6MWD: Distance walked during the 6-minute walk test; HR: Heart rate; Oxym: Oximetria; 7dVMCPM: 7 days vector magnitud counts per minute; SGRQ: Saint George Respiratory Questionnaire; IADL SCORE: Instrumental Activities Of Daily Living Scale; GAD7: General Anxiety Disorder-7 Questionnaire.

Report 6 (Confidence interval of other variables)

Table S9: Quality of life differences between groups in each evaluation, mean with 95% confidence intervals and p-value between baseline vs 8 weeks; 3-, and 6 months follow-up

	**CONTROL**			**INTERVENTION**			Difference				
	N	Mean	Std. Deviation	N	Mean	Std. Deviation	Mean	Srd. Error	95% Confidence Interval		p-value
									Lower	Upper	
GAD7 (BL-8W)	14	4.86	6.75	18	1.83	2.38	-3.02	1.71	-6.51	0.46	0.09
GAD7(BL-3M f.u)	13	3.77	4.76	16	3	5.59	-0.77	1.96	-4.78	3.24	0.70
GAD7 (BL-6M f.u)	8	4.5	7.8	15	2.87	5.72	-1.63	2.84	-7.54	4.27	0.57
SGRQ_Symptoms (BL-8W)	14	48.75	14.83	18	52.67	17.35	3.92	5.81	-7.95	15.79	0.51
SGRQ_Symptoms (BL-3M f.u)	13	47.9	20.33	16	47.59	16.36	-0.31	6.81	-14.28	13.65	0.96
SGRQ_Symptoms (BL-6M f.u)	8	49.53	15.37	15	43.54	19.08	-5.99	7.85	-22.31	10.33	0.45
SGRQ_Activity (BL-8W)	14	80.38	17.57	18	77.57	13.71	-2.81	5.52	-14.09	8.47	0.61
SGRQ_Activity (BL-3M f.u)	13	74.83	18.02	16	72.04	17.63	-2.79	6.65	-16.44	10.85	0.68
SGRQ_Activity (BL-6M f.u)	8	78.19	12.14	15	75.73	17.63	-2.46	7.01	-17.04	12.11	0.73
SGRQ_Impacts (BL-8W)	14	40.65	20.34	18	37.02	13.5	-3.63	5.99	-15.86	8.6	0.55
SGRQ_Impacts (BL-3M f.u)	13	36.41	16.5	16	37.33	15.61	0.93	5.98	-11.34	13.19	0.88
SGRQ_Impacts (BL-6M f.u)	8	35.2	14.59	15	35.55	12.38	0.35	5.76	-11.63	12.33	0.95
SGRQ_Total (BL-8W)	14	54.04	16.27	18	51.91	10.59	-2.13	4.76	-11.85	7.59	0.66
SGRQ_Total (BL-3M f.u)	13	49.96	13.65	16	49.56	11.85	-0.41	4.74	-10.12	9.31	0.93
SGRQ_Total (BL-6M f.u)	8	50.61	11.51	15	49.06	11.73	-1.56	5.1	-12.17	9.06	0.76
IADL SCORE (BL-8W)	14	1.14	1.03	18	0.78	0.94	-0.37	0.35	-1.08	0.35	0.30
IADL SCORE (BL-3M f.u)	13	0.85	0.9	16	0.81	0.98	-0.03	0.35	-0.76	0.69	0.92
IADL SCORE (BL-6M f.u)	8	1	0.93	15	0.87	1.25	-0.13	0.5	-1.18	0.91	0.79

Table S10: Seven days pedometry and vector magnitude counts per minute differences between groups at each follow-up, mean with 95% confidence intervals and p value between baseline vs 8 weeks; 3-; and 6 months follow-up.

	CONTROL			INTERVENTION			Difference				
	N	Mean	Std. Deviation	N	Mean	Std. Deviation	Mean	Srd. Error	95% Confidence Interval		p-value
									Lower	Upper	
7 days pedometry (BL-8W)	13	11985	10783	18	9000	4242	-2985	2788	-8686	2717	0.29
7 days pedometry (BL-3M f.u)	12	10928	10304	16	7319	4984	-3609	2940	-9652	2433	0.23
7 days pedometry (BL-6M f.u)	7	9384	7068	14	7661	3986	-1723	2390	-6725	3279	0.48
7 d VMCPM (BL-8W)	13	420	283	18	304	133	-117	76	-272	39	0.14
7 d VMCPM (BL-3M f.u)	12	431	337	16	260	144	-171	94	-363	22	0.08
7 d VMCPM (BL-6M f.u)	7	376	205	14	453	635	78	249	-443	599	0.76

Report 7 (Non inferiority 8 weeks vs 3- and 6 months follow-up)

Extra analysis of the differences between the follow-up.

To analyze 3 months and 6 months follow-up comparing the groups after the intervention treatment an independent test of the differences within groups considering the differences between the results over time were calculated.

Table S11. Three months follow-up difference after treatment.

After treatment compared 3 months follow-up					Differences Between Treatments		95% Confidence Interval of the Difference		p-value
8W-3M f.u	Treatment	n	Mean	Std. Deviation	Mean Difference	Std. Error Difference	Lower	Upper	
6MWT	Intervention	16	0.44	31.00	-11.03	15.79	-44.05	21.99	0.493
	Control	13	11.47	49.60					
GAD7	Intervention	16	-1.13	3.96	-2.59	2.01	-6.70	1.53	0.208
	Control	13	1.46	6.73					
SGRQ_Symtoms	Intervention	16	2.85	12.63	3.87	5.74	-7.91	15.65	0.506
	Control	13	-1.01	18.23					
SGRQ_Activity	Intervention	16	5.29	13.02	0.67	4.89	-9.36	10.71	0.891
	Control	13	4.61	13.20					
SGRQ_Impacts	Intervention	16	-1.33	16.15	-4.87	5.98	-17.15	7.41	0.423
	Control	13	3.54	15.87					
SGRQ_Total	Intervention	16	1.37	10.02	-1.74	4.04	-10.03	6.55	0.670
	Control	13	3.11	11.74					
IADL	Intervention	16	0.44	31.00	-11.03	15.79	-44.05	21.99	0.493
	Control	13	11.47	49.60					

Table S12. Six months follow-up difference after treatment.

After treatment compared with 6 months follow-up					Differences Between Treatments		95% Confidence Interval of the Difference		p-value
8W-6M f.u	Treatment	n	Mean	Std. Deviation	Mean Difference	Std. Error Difference	Lower	Upper	
6MWT	Intervention	15	8.53	50.53	-27.72	23.02	-75.60	20.16	0.242
	Control	8	36.25	56.48					
GAD7	Intervention	15	-1.33	5.15	-1.96	2.28	-6.70	2.78	0.400
	Control	8	0.63	5.32					
SGRQ_Symtoms	Intervention	15	7.63	19.04	9.48	7.29	-5.67	24.63	0.207
	Control	8	-1.85	10.27					
SGRQ_Activity	Intervention	15	0.59	11.54	0.47	5.30	-10.54	11.49	0.930
	Control	8	0.12	13.15					
SGRQ_Impacts	Intervention	15	-0.20	9.96	-7.09	7.02	-22.91	8.73	0.338
	Control	8	6.89	18.47					
SGRQ_Total	Intervention	15	1.34	9.56	-2.05	4.76	-11.94	7.84	0.671
	Control	8	3.38	13.08					
IADL	Intervention	15	8.53	50.53	-27.65	23.03	-75.55	20.24	0.243
	Control	8	36.19	56.52					

Report 8 (Exercise Time, Adherence and Patient Satisfaction in the Telerehabilitation with VAPA group)

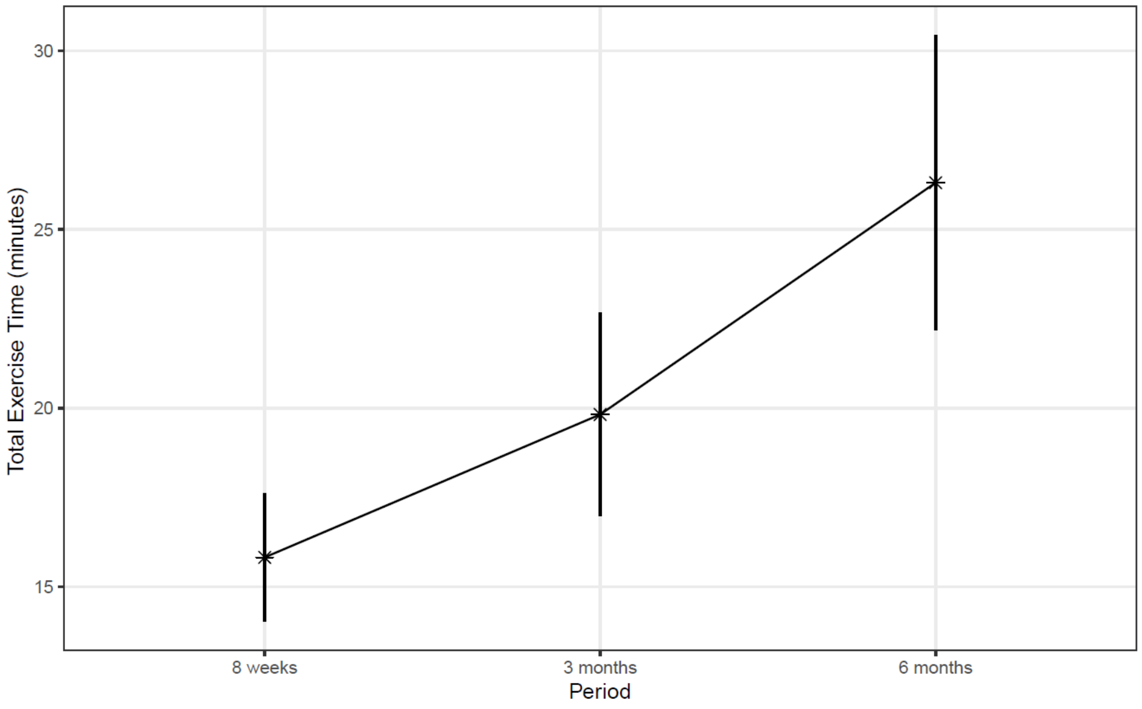

Figure S14: Exercise time by patients in the TR with VAPA. Data is shown as the average time (min) per exercise session (excluding pause time).

Table S13: Patient adherence and satisfaction shown as the training time expected vs performed and patient satisfaction from baseline to follow-up after 8 months.

		Adherence			Satisfaction	
		Exercise minutes			Answers	Mean ± SD *
		Expected	Trained			
Patients	Period	Number		%		
21	0-8 W	480	394	82		
10	8W-3M f.us	720	646	90	465	4.27 ± 0.77
5	3-6M f.us	720	614	85		

*on the 5-point scale average (1 very unsatisfied - 5 very satisfied)
